# Physicochemical Parameters of Real Wastewater Originating from a Plant Protection Products Factory and Modification of the QuEChERS Method for Determination of Captan

**DOI:** 10.3390/molecules24122203

**Published:** 2019-06-12

**Authors:** Ewa Szpyrka, Maciej Thomas, Magdalena Podbielska

**Affiliations:** 1 Department of Analytical Chemistry, Faculty of Biotechnology, University of Rzeszow, Pigonia 1, 35-310 Rzeszow, Poland; magdapodbiel@gmail.com; 2 Chemiqua Company, Skawinska 25/1, 31-066 Krakow, Poland; biuro@chemiqua.pl

**Keywords:** QuEChERS, captan, pesticides, industrial wastewater, gas chromatography

## Abstract

The aim of this study was the modification and application of the QuEChERS method for the preparation and purification of samples in order to determine the level of captan in real wastewater originating from a plant protection products factory which was characterized by a significant content of organic substances [Chemical Oxygen Demand (COD) = 856 ± 128 mg O_2_/L and Total Organic Carbon (TOC) = 62 ± 9 mg/L]. The optimization of the method consisted of the selection of solvents used for the extraction of captan from wastewater and also sorbents used to purify the extracts by the dispersion of a solid phase extraction technique (dSPE). Two steps were used: extraction and clean-up. In the extraction step, acetonitrile was replaced by anacetonitrile:acetone mixture. In the clean-up step by the dSPE, five sorbents were tested: Florisil^®^, aluminum oxide (Al_2_O_3_), zirconium oxide (ZrO_2_), silicon oxide (SiO_2_) and PSA (primary and secondary amine). Concentrations of captan in wastewater extracts were determined by gas chromatography (GC) combined with electron capture detection (μECD). The best recovery parameters and precision of the method were obtained for samples purified using ZrO_2_ (recovery 98% and precision expressed as relative standard deviation RSD 8%) and Florisil^®^ (recovery 96%, RSD 9%). Limits of detection (LOD) and quantification (LOQ) for determination of captan in diluted extract of wastewater were 0.003 and 0.01 mg/L, respectively. Matrix effects were in the range of −69% to −44% for samples purified by ZrO_2_ and Florisil^®^, respectively. The modified and optimized method was applied for fast and simple determination of captan levels in real industrial wastewater samples, in which the concentration of captan in diluted extract was determined to be 4.0 ± 0.3 mg/L.

## 1. Introduction

Plant protection products are a mixture of various substances, i.e., active substances used to control pests, diseases and weeds, adjuvants which improve adhesion to plants and numerous functional additives with emulsifying, wetting, anti-freezing, anti-scaling, dispersing or anti-foaming properties [1,2]. These substances are responsible for ensuring the appropriate application properties of products. As a result of their use in technological processes, these substances contaminate the wastewater, significantly determining their composition and physicochemical properties even though they often have a negative impact on the efficiency of treatment processes used in factory sewage treatment plants. The presence of many organic substances in wastewater with different chemical structures may also be the cause of many analytical problems when determining the content of pesticides in the wastewater in question.

**Figure 1:**
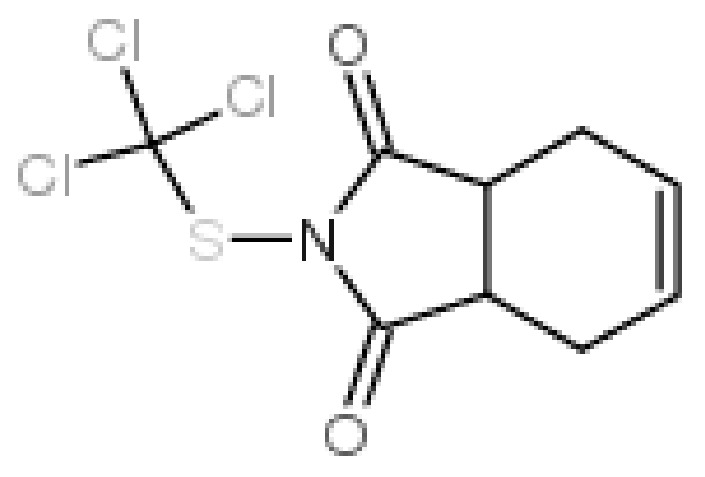
Chemical structure of captan (N-{trichloromethylthio}cyclohex-4-ene-1,2-dicarboximide).

Captan (IUPAC name: N-{trichloromethylthio}cyclohex-4-ene-1,2-dicarboximide, Figure 1) is a very popular active substance used in many European countries as a fungicide to protect several agricultural products, including fruit, i.e., apples, apricots, blueberries, blackberries, cherries, grapes, raspberries, nectarines, plums, peaches, and almonds, grasses, and roses [3,4]. In Poland, for instance, there are 34 plant protection products which contain captan [5], produced in the form of powders, granules, or concentrates for dissolving in water.

The Quick, Easy, Cheap, Effective, Rugged, Safe (QuEChERS) method employing dispersive Solid Phase Extraction (dSPE) is a simple and fast procedure to clean-up food samples for pesticide analysis. The method was designed for the extraction and clean-up of samples for the determination of pesticides in fruit and vegetables [6]. The QuEChERS method is also the standard sample preparation procedure in the EU prior to determination of pesticide residues using gas chromatography–mass spectrometry (GC–MS) and/or liquid chromatography–tandem mass spectrometry (LC–MS/MS) [7]. Nowadays, it has been adapted for the preparation of samples of different matrices, e.g., meat, milk, honey, oil, soil, and human fluids and to determine not only pesticides, but also various other classes of chemicals, e.g., volatile organic compounds (VOCs), polycyclic aromatic hydrocarbons (PAHs), mycotoxins, pharmaceuticals, veterinary drugs, flame retardants, and forensic samples [8,9]. Only a few publications, however, describe the adaptation of the QuEChERS method for the determination of pesticides in drainage waters [10], wastewater [11,12], or sewage sludge [13].

As stated above, the aim of this study was to evaluate selected physicochemical parameters of real wastewater originating from a plant protection products factory and modification and application of the QuEChERS method of preparation and purification of samples in order to determine the level of captan in wastewater by gas chromatography (GC) combined with electron capture detection (µECD).

## 2. Results

The wastewater originating from the same plant protection products factory was analysed for selected physicochemical parameters. All analyses were performed in accordance with ISO procedures (see Table 1). The analysed industrial wastewater (A-sample and B-sample) was slightly alkaline (pH = 7.5 and 7.6, respectively) and was characterized by a significant content of organic compounds (COD = 830 and 856 mg O_2_/L, respectively). The TOC values were 58 and 62 mg/L for the A- and B-samples, respectively. Furthermore, both analysed wastewater samples contained small quantities of phosphorus (0.5 and 0.6 mg/L) and nitrogen (2.00 and 2.20 mg/L). In addition, the wastewater also contained small amounts of heavy metals, i.e., copper, iron, nickel, and zinc, as well as aluminum (0.10 ± 0.01, 0.040 ± 0.004, 0.050 ± 0.005, 1.30 ± 0.13, and 0.24 ± 0.02 mg/L, respectively). The A-sample (“blank”—without captan) was used in the validation step, while the B-sample (“real” sample) was used to determine the level of captan using the described method.

Our modification of the QuEChERS method for the determination of captan in wastewater was divided into two steps: extraction and clean-up. In the extraction step, instead of 10 mL acetonitrile, a 10 mL acetonitrile:acetone mixture (1:1 *v/v*) was added to 5 mL of the wastewater sample. In the case when only acetonitrile was added to a sample, three layers were formed and further analysis was impossible. Our modification facilitates the creation of a homogenous solution. In the clean-up step using the dSPE, five sorbents were tested: Florisil^®^, aluminum oxide(Al_2_O_3_), zirconium oxide (ZrO_2_), silicon oxide (SiO_2_) and PSA (primary and secondary amine). Each sample was analysed in six replicates.

Next, validation was conducted to assess the following parameters: selectivity, linearity, matrix effects (MEs), accuracy (trueness and repeatability with intra-day and inter-day tests), limits of detection (LOD) and quantification (LOQ). The validation of the method was carried out using wastewater collected from the factory (A-sample) that was previously subjected to pesticide residue analysis. For the blank extract and a spiked blank extract, no interfering peaks were noticed; therefore, the method is selective for captan determination. Identical retention times of captan for solvent-matched and matrix-matched standards were stated. Linearity was studied by analysing solvent-matched and matrix-matched standards at five concentration levels: 0.01; 0.05; 0.5; 1.0 and 5.0 mg/L, in three replicates for each level. A calibration curve equation in the solvent was y = 5795 x − 367 and the correlation coefficient (R) was equal to 0.999. Calibration curve equations for samples cleaned-up by sorbents had good linearity in the same range 0.01–5.0 mg/L and R was in the range 0.996–0.997 (see Table 2).

MEs ranged from −69% to −44% for samples cleaned-up by ZrO_2_ and Florisil^®^, respectively (Table 2). The wastewater caused the suppression of the detection system, therefore, quantification was done to matrix-matched standards. The accuracy of the method was estimated by recovery studies. Recoveries were determined in six repetitions at two spiking levels: 0.1 mg/L (which corresponds to LOQ for the diluted extract) and 50 mg/L. The samples of blank wastewater were spiked with a known amount of captan prior to the initiation of the sample preparation procedure. Precision (repeatability with intra-day and inter-day tests) was expressed as the percentage of relative standard deviation (%RSD).

**Figure 2:**
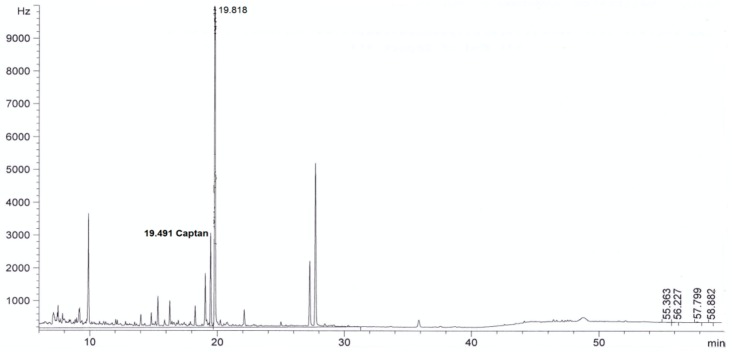
The chromatogram (GC-μECD) of industrial wastewater without the clean-up step using the dispersive solid phase extraction technique (dSPE).

**Figure 3:**
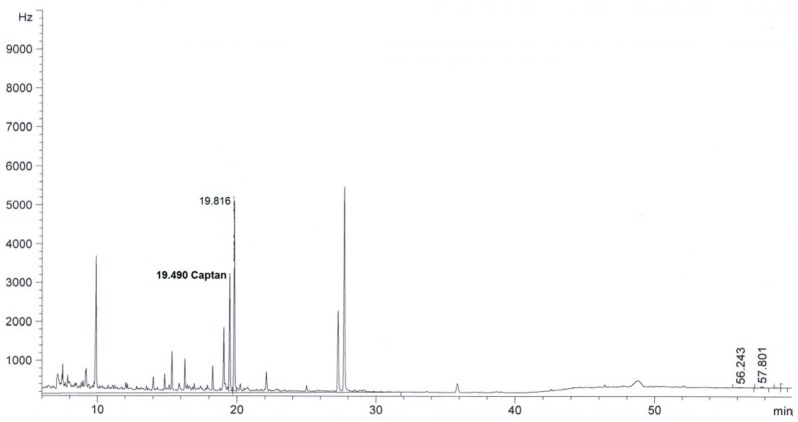
The chromatogram (GC-μECD) of industrial wastewater with the clean-up step using the dSPE (1 g Al_2_O_3_).

**Figure 4:**
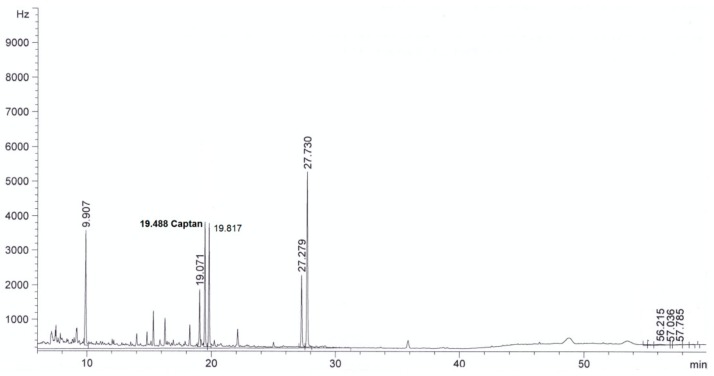
The chromatogram (GC-μECD) of industrial wastewater with the clean-up step using the dSPE (0.15 g SiO_2_).

**Figure 5:**
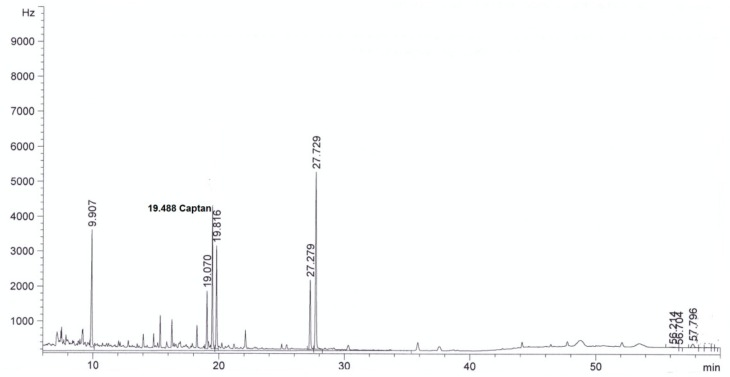
The chromatogram (GC-μECD) of industrial wastewater with the clean-up step using the dSPE [0.15 g primary and secondary amine (PSA)].

**Figure 6:**
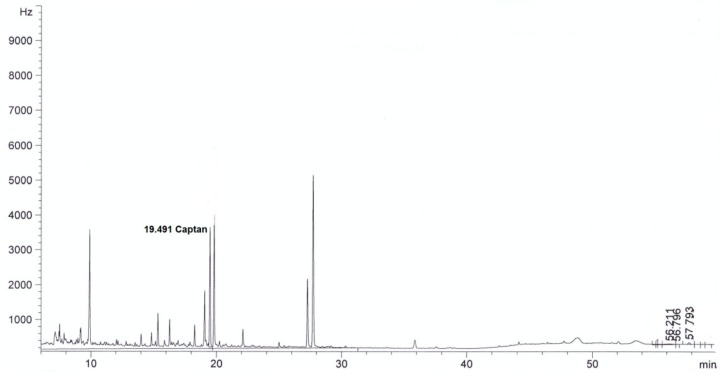
The chromatogram (GC-μECD) of industrial wastewater with the clean-up step using the dSPE (1 g ZrO_2_).

**Figure 7:**
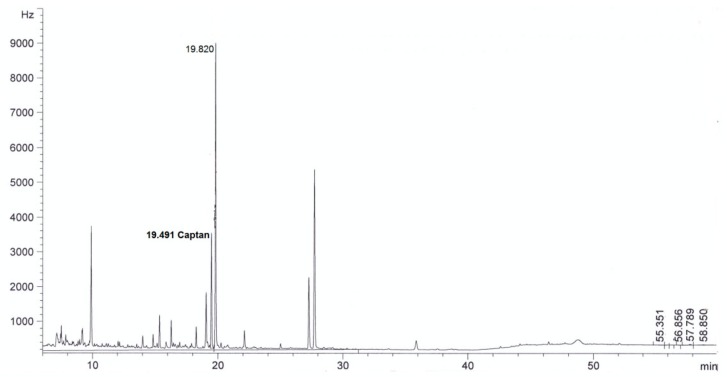
The chromatogram (GC-μECD) of industrial wastewater with the clean-up step using the dSPE (1 g Florisil^®^).

For all sorbents, good validation parameters were obtained, which are in line with criteria for pesticide residues methods: RSD below or equal to 20% and recovery from 70% to 120% [24] (see Table 2). The best recovery parameters and precision of the method were obtained for samples purified using ZrO_2_ (recovery 98% and precision expressed as RSD 8%) and Florisil^®^ (recovery 96%, RSD 9%). Better purification of the extract was obtained in the case of ZrO_2_ compared to Florisil^®^, which can be seen from the chromatograms by the reduction in interferences (see Figure 2, Figure 3, Figure 4, Figure 5, Figure 6 and Figure 7). For determination of captan in wastewater samples (characterized by parameters in Table 1) the best sorbent for clean-up is ZrO_2_.

The LOD of captan in 10-fold diluted extract of wastewater was calculated as 0.003 mg/L, based on the noise level in the chromatograms at a signal-to-noise ratio (S/N) of 3:1. The LOQ 0.01 mg/L was set at the lowest spiking level for which satisfactory recovery and precision parameters were achieved.

After that, concentrations of captan in wastewater extracts were determined by gas chromatography (GC) combined with electron capture (μECD) detection using an Agilent Technologies 7890A system. Quantification was performed in the linear range of detector response to captan (0.01–5.0 mg/L). The modified and optimized method was applied for the fast and simple determination of captan in real wastewater samples (B-sample). The 10-fold diluted extract of wastewater from the factory contained captan in the concentration of 4.0 ± 0.3 mg/L.

## 3. Discussion

Captan is an active ingredient of a popular fungicide used worldwide [4]. It belongs to the dicarboximide group of pesticides. Its solubility in water is 5.2 mg/L (at 25 °C), 38 g/L (at 20 °C) in acetone [4] and 36 g/L in acetonitrile [25]. This pesticide is determined by GC or GC mass spectrometry and it is not amenable to LC–MS/MS [26,27].

The physicochemical properties of wastewater are varied for different plant protection products factories and depend on many technical and technological parameters. For wastewater used in this research, the COD-values, (830 and 856 mg O_2_/L) were relatively low compared to the value obtained by other researchers (11,400 mg/L) [28]. In the other studies, authors reported the following values for pH, COD, Total Dissolved Solids (TDS): 12–14, 6000–7000 mg O_2_/L, 12,000–13,000 mg/L, respectively [29]. In this study, values of pH 7.5 and 7.6, COD 830 and 856 mg O_2_/L and TDS 430 and 418 mg/L were obtained. As other authors also emphasize, the pesticides’ wastewater depicts a wide variation in the wastewater characteristics. It depends not only on the types of agrochemical manufactured but also on the raw materials utilized [29].

QuEChERS is a very popular method for sample preparation for pesticide analysis, especially for food products. Nowadays, it is adapted for the preparation of analysis samples for different matrices and the determination of not only pesticides, but also various chemicals [8,9]. Only a few publications, however, discuss the adaptation of the QuEChERS method for the determination of pesticides in agricultural drainage waters [10], food wastewater [11], industrial wastewater [12], and sewage sludge from an agro-food industry [13]. These methods concern determinations of pesticides from different chemical classes but none of these publications mention captan level determination. For the determination of captan levels in groundwater, a method based on voltammetry was published, in which the recovery of substances was equal to 98.9% with LOD and LOQ 85 picomol (pM) and 280 pM, respectively [30]. On the other hand, Soares et al. [31] applied a vortex-assisted Matrix Solid Phase Dispersion with determination by gas chromatography coupled to mass spectrometry for the analysis of captan and other pesticides in drinking water treatment sludge with recoveries in the 93–106% range. Additionally, in this case, the LOQ for captan was 0.1 mg/kg. A liquid–liquid extraction with organic solvents: hexane/ether solvent [32] or petroleum ether [33] is quite a popular method for captan extraction from water samples. The U.S. Environmental Protection Agency (EPA) developed the solid phase extraction (SPE) method followed by gas chromatography/mass spectrometry (GC–MS) for identification and quantitation of captan levels in drinking water [34]. In the case of the determination of pesticides in drinking water, however, lower LOQ are required taking into account the health safety of consumers.

The modified and optimized method can be applied for fast and simple determination of captan levels in real wastewater samples and could be adopted for the determination of other lipophilic chemicals with similar properties to captan.

## 4. Materials and Methods

### 4.1. Materials

The captan standard was purchased from Dr. Ehrenstorfer (Augsburg, Germany). All reagents were of sufficiently high purity for GC analysis. Acetonitrile, acetone, and petroleum ether were purchased from Honeywell Specialty Chemicals Seelze GmbH (Seelze, Germany). Anhydrous magnesium sulphate, sodium chloride, and tri-sodium citrate dihydrate were from Chempur (Piekary Slaskie, Poland). Sodium citrate dibasic sesquihydrate was purchased from Sigma-Aldrich (Steinheim, Germany). As for the sorbents, PSA was purchased from Supelco (Bellefonte, PA, USA), Al_2_O_3_, ZrO_2_, SiO_2_ were from POCH (Gliwice, Poland) and Florisil^®^ was from Sigma-Aldrich (St. Louis, MO, USA).

### 4.2. Methods

**Figure 8:**
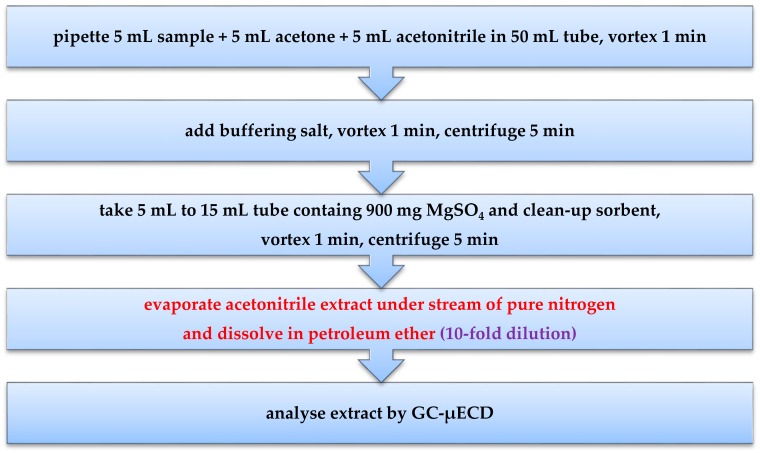
Scheme of real industrial wastewater sample preparation.

The wastewater from a plant protection products factory (A-sample and B-sample) was analysed for selected physicochemical parameters. All analysis was conducted according to ISO procedures (see Table 1). Modification of the QuEChERS method [7], which is used for pesticide residues analysis in food of plant origin was carried out to adapt it to wastewater samples. In the original method, the mass of a sample used ranges from 2 to 10 g, depending on the type of sample. In the case of samples with low water content, before extraction, water was added to samples. Then, 10 mL of acetonitrile was added before the extraction step. In our study, in the extraction step, instead of 10 mL acetonitrile, 10 mL acetonitrile:acetone mixture (1:1 *v/v*) was added, along with 5 mL of the wastewater sample, into a 50 mL disposable polypropylene centrifuge tube. In the case when only acetonitrile was added to the sample, three layers were formed and further analysis was impossible. Our modification allows for the creation of a homogenous solution. The content of the centrifuge tube was vortexed by mixer (BenchMixer^TM^, Benchmark, Edison, NJ, USA) for 1 min. Then, a mixture of salts containing 4 g of anhydrous magnesium sulfate, 1 g of sodium chloride, 1 g of trisodium citrate, and 0.5 g of disodium hydrogen citrate sesquihydrate were added. The content was vortexed by a mixer (BenchMixer^TM^, Benchmark) for 1 min and then centrifuged for 5 min at 3000 rpm (MPW-350R, MPW MED. INSTRUMENTS, Warszawa, Poland). A quantity of 5 mL of the acetonitrile phase was transferred to a 15-mL disposable polypropylene centrifuge tube that contained 900 mg of anhydrous magnesium sulfate and the appropriate sorbent for clean-up. In this step, five sorbents were tested: Florisil^®^ (1 g), Al_2_O_3_ (1 g), ZrO_2_ (1 g), SiO_2_ (0.15 g), and PSA (0.15 g). The content of the tube was vortexed for 1 min by mixer (BenchMixer^TM^, Benchmark) and centrifuged for 5 min at 3000 rpm (MPW-350R, MPW MED. INSTRUMENTS). A total of 1 mL of obtained extract was transferred to a volumetric flask, acetonitrile extract was evaporated to dryness under a stream of pure nitrogen and then dissolved in 10 mL of petroleum ether (Chempur) (see Figure 8). Six replicates of each sample were analysed.

Furthermore, the concentrations of captan in wastewater extracts were determined by gas chromatography (GC) combined with electron capture (μECD) detection (7890A system, (Agilent Technologies, Palo Alto, CA, USA). A quantity of 2 µL of samples were injected in splitless mode on a HP-5 MS Ultra Inert/30 m × 0.25 mm I.D. × 0.25-µm column. The carrier gas was helium with a flow 3.7 mL/min. The temperature of the injector and detector were 250 and 300 °C, respectively. The Agilent Technologies ChemStation, Rev. B04.03-SP2(108) package was used as software for processing the analysis results [35].

Validation of the proposed method was conducted to assess the following parameters: selectivity, linearity, MEs, accuracy (trueness and repeatability with intra-day and inter-day tests (over the course of 6 days), limits of detection (LOD) and quantification (LOQ). The validation of the method was carried out using wastewater collected from the factory (without captan, A-sample) that had been previously subjected to pesticide residue analysis. Blank extract of wastewater and a spiked blank extract were injected into the GC to verify the retention time of captan and the interfering peak for selectivity.

Linearity was studied by analysing solvent-matched and matrix-matched standards at five concentration levels: 0.01, 0.05, 0.5, 1.0 and 5.0 mg/L. Three replicates were done for each point of the calibration curve. Calibration curve equations and correlation coefficients (R) were calculated using the ChemStation software which controls the GC system.

For estimating the matrix effects, matrix-matched standards were prepared at five concentration levels: 0.01, 0.05, 0.5, 1.0 and 5.0 mg/L. Three replicates were done for each point of the calibration curve. MEs were calculated as follows: %ME = [(Slope of the matrix-matched calibration−Slope of reagent-only calibration)/Slope of reagent-only calibration] × 100 [35,36].

Method accuracy was estimated by recovery studies. Recoveries were determined in six repetitions at two spiking levels: 0.1 mg/L (which corresponds to LOQ for the diluted extract) and 50 mg/L. The samples of blank wastewater were spiked with a known amount of captan prior to the initiation of the sample preparation procedure. Precision (repeatability with intra-day and inter-day tests) was expressed as the percentage of RSD (% RSD). The LOD of captan was calculated based on the noise level in the chromatograms at S/N of 3:1. An LOQ value of 0.01 mg/L was set at the lowest spiking level for which satisfactory recovery and precision parameters were achieved. The LOQ is consistent with Polish law for entering sewage into sewage devices with organochlorine volatile compounds—permitted value of 1.5 mg Cl/L [37].

Validation parameters were determined for each sorbent (see Table 2). The modified and optimized method was applied for fast and simple determination of captan levels in the real wastewater sample (B-sample).

## 5. Conclusions

The modified and optimized QuEChERS method presented in this study can be applied for the fast and simple determination of captan levels in real wastewater samples with high contents of organic substances (as determined by the chemical oxygen demand and total organic carbon). The results obtain by the described method can be considered correct because it is characterized by good selectivity, linearity, accuracy, LOD and LOQ. For accurate quantification of the captan content in wastewater, matrix-matched standards should be used.

## Figures and Tables

**Table 1:** Selected physicochemical parameters of real wastewater that originated from a plant protection products factory.

Parameter	Method	Result	
		A-Sample	B-Sample
Captan, mg/L	This method	<0.003 in diluted extract	4.0 ± 0.3 in diluted extract
pH	PN-EN ISO 10523:2012 [14]	7.5 ± 0.1	7.6 ± 0.1
Electrical conductivity, µS/cm	PN-EN 27888:1999 [15]	900 ± 45	850 ± 43
Salinity, mg NaCl/L	PN-EN 27888:1999 [15]	430 ± 22	418 ± 21
Color, mg Pt/L	PN-EN ISO 7887:2012 [16]	50 ± 10	52 ± 10
Turbidity, NTU	PN-EN ISO 7027:2003 [17]	880 ± 90	900 ± 90
Chloride, mg/L	PN-ISO 9297:1994 [18]	75 ± 8	80 ± 8
Sulfates, mg/L	PN-ISO 9280:2002 [19]	95 ± 10	108 ± 11
COD, mg O_2_/L	PN-ISO 15705:2005 [20]	830 ± 120	856 ± 128
TOC, mg/L	PN-EN 1484:1999 [21]	58 ± 6	62 ± 9
Total phosphorus, mg/L	PN-EN ISO 11885:2009 [22]	0.5 ± 0.08	0.60 ± 0.06
Total nitrogen, mg/L	PN-EN ISO 12260:2004 [23]	2.00 ± 0.30	2.20 ± 0.33
Copper, mg/L	PN-EN ISO 11885:2009 [22]	0.10 ± 0.01	0.10 ± 0.01
Iron, mg/L	PN-EN ISO 11885:2009 [22]	0.060 ± 0.006	0.040 ± 0.004
Nickel, mg/L	PN-EN ISO 11885:2009 [22]	0.050 ± 0.005	0.050 ± 0.005
Zinc, mg/L	PN-EN ISO 11885:2009 [22]	1.20 ± 0.12	1.30 ± 0.13
Aluminum, mg/L	PN-EN ISO 11885:2009 [22]	0.20 ± 0.02	0.24 ± 0.02

**Table 2:** Validation parameters for captan determination with the QuEChERS method with different sorbents in the dSPE method.

Sorbent	Matrix-Match Calibration Curve Equation	Correlation Coefficient (R)	ME (%)	Recovery (%) at Spiking Level 0.1 mg/L	Recovery (%) at Spiking Level l50 mg/L	Overall Recover (%)	Intra-Day Relative Standard Deviation (%)	Inter-day Relative Standard Deviation (%)
Florisil^®^	y = 3238 x − 381	0.997	−44	95	97	96	9	10
Al_2_O_3_	y = 2333 x − 331	0.996	−60	89	92	91	10	12
ZrO_2_	y = 1796 x − 237	0.996	−69	93	103	98	8	10
SiO_2_	y =3093 x − 399	0.997	−47	71	76	74	16	19
PSA	y =1904 x − 247	0.996	−67	95	89	92	18	20
